# Large-Scale Transportation Network Congestion Evolution Prediction Using Deep Learning Theory

**DOI:** 10.1371/journal.pone.0119044

**Published:** 2015-03-17

**Authors:** Xiaolei Ma, Haiyang Yu, Yunpeng Wang, Yinhai Wang

**Affiliations:** 1 School of Transportation Science and Engineering, Beijing Key Laboratory for Cooperative Vehicle Infrastructure, Systems, and Safety Control, Beihang University, Beijing, China; 2 Jiangsu Province Collaborative Innovation Center of Modern Urban Traffic Technologies, SiPaiLou #2, Nanjing, China; 3 Department of Civil and Environmental Engineering, University of Washington, Seattle, Washington, United States of America; Universidad de Zarazoga, SPAIN

## Abstract

Understanding how congestion at one location can cause ripples throughout large-scale transportation network is vital for transportation researchers and practitioners to pinpoint traffic bottlenecks for congestion mitigation. Traditional studies rely on either mathematical equations or simulation techniques to model traffic congestion dynamics. However, most of the approaches have limitations, largely due to unrealistic assumptions and cumbersome parameter calibration process. With the development of Intelligent Transportation Systems (ITS) and Internet of Things (IoT), transportation data become more and more ubiquitous. This triggers a series of data-driven research to investigate transportation phenomena. Among them, deep learning theory is considered one of the most promising techniques to tackle tremendous high-dimensional data. This study attempts to extend deep learning theory into large-scale transportation network analysis. A deep Restricted Boltzmann Machine and Recurrent Neural Network architecture is utilized to model and predict traffic congestion evolution based on Global Positioning System (GPS) data from taxi. A numerical study in Ningbo, China is conducted to validate the effectiveness and efficiency of the proposed method. Results show that the prediction accuracy can achieve as high as 88% within less than 6 minutes when the model is implemented in a Graphic Processing Unit (GPU)-based parallel computing environment. The predicted congestion evolution patterns can be visualized temporally and spatially through a map-based platform to identify the vulnerable links for proactive congestion mitigation.

## Introduction

Traffic congestion costs billions of dollars in each year due to lost time, wasted fuel, excessive air pollution, and reduced productivity. The 2012 Urban Mobility Report indicates that the annual average delay per person was 38 hours in 2011 for the 498 surveyed urban areas, which is equivalent to a 238% increase compared to that in 1982. Traffic congestion incurred a total of 5.5 billion hours of travel delays and 2.9 billion gallons of extra fuel consumption in 2009, which corresponds to a congestion cost of 121 billion dollars [1].

Diagnosing congestion onset and predicting congestion evolution patterns are considered strategic countermeasures to locate traffic bottlenecks and adopt proactive measures for congestion mitigation. Many research efforts have been made to achieve these goals [2, 3]. However, most of previous studies tend to view congestion spots separately in a small-scale network. As mentioned by Yang[4], the number of network links for almost all the existing traffic congestion prediction methods do not exceed 100. In addition, these studies rely on either mathematical equations or simulation techniques to depict the network congestion evolution. This often results in suboptimality since transportation activities involve human factors which are difficult to represent or model accurately using mathematics-driven approaches. Previous network-wide congestion studies mainly resort to either complex network theory [5–11] or visualization techniques [12] to understand the evolution of network-wide traffic congestion. In complex network theory, transportation networks can be abstracted as scale-free networks [9], and traffic flow dynamics over the network are generated based on the power law distribution [11] However, these assumptions are not always adherent to the reality, and lack sufficient traffic sensor data to validate their findings. Visualization techniques can intuitively display the spatial and temporal distribution of network congestion through a map-based platform, but are incapable of explaining the mechanism of congestion generation and predicting future trend of congestion evolution.

Over the past decades, tremendous traffic sensors have been deployed on the existing freeway networks, generating a huge amount of data at relatively high time resolutions. The increasing availability of network data makes it possible to simultaneously examine traffic flows on a large-scale roadway network and observe the evolution of congestion on that network through data mining techniques. Transportation network consists of thousands of links with changing traffic condition over time. This is equivalent to a high-dimensional space where congestion prorogates temporally and spatially. This is challenging to model using traditional data mining approaches due to the curse of dimensionality: when the input dimensionality increases, the required training data grow exponentially [13]. The recent emergence of deep learning theory can address the curse of dimensionality issue through distributed representations, and thereby holds great promise in learning high-dimensional features with tremendous data. Compared to those shallow learning architectures, deep learning is able to model complex non-linear phenomenon using distributed and hierarchical feature representation [14, 15], and has received numerous success in the domain of computer vision, speech recognition, natural language processing and music composition [16]. Deep learning theory began to exhibits its superiority of predicting traffic flow over a single road segment [14]. However, to the best of our knowledge, no research has been conducted to apply deep learning theory into large-scale transportation network modeling and analysis. To assist transportation professionals in congestion diagnosis and operational strategy assessment, this study aims at developing a data-driven and network-wide congestion analysis paradigm based on traffic sensor measurements using deep learning theory.

The remainder of this paper is organized as follows: Section 2 presents a methodological framework to model network-wide traffic congestion, where GPS data are collected to depict the roadway traffic condition and a deep learning architecture Recurrent Neural Network and Restricted Boltzmann Machine (RNN-RBM) is introduced to predict the temporal-spatial congestion evolution pattern. To validate the effectiveness of the proposed algorithm, a numerical experiment with a city roadway network of over 500 links is undertaken in Section 3. The prediction results are demonstrated through a GIS platform to observe the future congestion evolution, followed by a sensitivity analysis with various congestion speed thresholds. In addition, a comparative study with conventional neural network and support vector machine methods is conducted to demonstrate the advantage of the proposed algorithm. Finally, discussion and conclusion are made in the last section.

## Methodology

Traffic condition on each link is classified into a binary state, where 1 represents congested and 0 represents uncongested. The link traffic condition changes over time, and essentially becomes a binary sequence with varying length. Considering the size of a typical transportation network, the network-wide congestion pattern is equivalent to a high-dimensional matrix arranged in time (number of time steps) and space (number of links). The key of network-wide traffic congestion evolution prediction model is to understand how each matrix element evolves spatially and temporally. The initial step is to identify the traffic condition of each link using taxi GPS data. Missing and erroneous data will be reviewed and corrected to ensure satisfying quality. Finally, processed GPS data are aggregated into varying time intervals for representing network-wide traffic patterns. Inspired by polyphonic music generation and transcription approach [17](Boulanger-Lewandowski, 2012), a combination of Restricted Boltzmann Machine (RBM) and Recurrent Neural Network (RNN) is then utilized to learn the high-dimensional traffic congestion patterns. To fully take advantages of Graphics Processing Unit (GPU)-based parallel computing architecture, the proposed algorithm is implemented in the NVIDIA Compute Unified Device Architecture (CUDA) environment to accelerate the model learning procedure.

### Transportation Network Representation

A GPS-equipped taxi can record its location and timestamp information, and the travel speed can be directly measured. For each link *n*, the average speed at time *t* is denoted as$c_{n}^{t}$, If the average speed is lower than a threshold (20 km/hour), the link is considered congested, and thereby is set as 1. Due to the sampling error, GPS-equipped taxis may not fully travel along the entire network at any time. For those links without GPS data, vehicle speed is calculated by using the historical records for the same link. Based on the above discussion, traffic congestions for a network with *N* links within *T* time intervals can be expressed as:

$$\left[\begin{array}{cccc} c_{1}^{1} & c_{1}^{2} & ... & c_{1}^{T}\\ c_{2}^{1} & c_{2}^{1} & ... & c_{2}^{T}\\ \vdots & \vdots & ... & \vdots \\ c_{N}^{1} & c_{N}^{2} & ... & c_{N}^{T} \end{array}\right]$$ (1)

Where $c_{n}^{t}$ represents the traffic congestion condition on *n*th link at time *t*, which is a binary value. The next step is to predict the elements in each row (link). This can be treated as the single sequence learning problem. When multiple rows (links) are predicted simultaneously, this is known as the high-dimensional sequence learning problem. A deep learning architecture with temporal processing capabilities is desired.

### RNN-RBM: A deep learning architecture for high-dimensional temporal sequence prediction

#### Restricted Boltzmann Machine (RBM)

**Fig 1:**
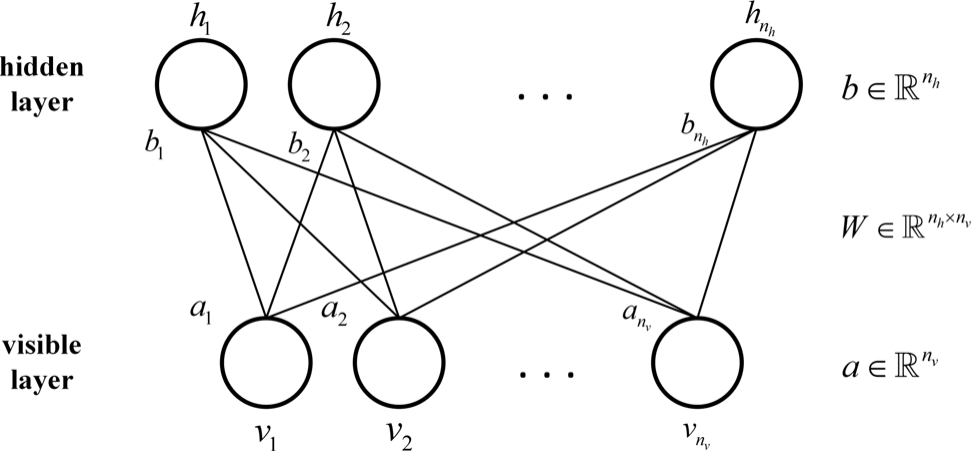
RBM Structure.

Restricted Boltzmann Machine (RBM) is an energy based model that includes one visible layer and one hidden layer [15]. As demonstrated in Fig. 1, units in both visible layer and hidden layer are mutually connected.

In Fig. 1, *n*_*v*_ and n_h_ respectively represent the number of units in hidden layer and visible layer. $v={\left(v_{1},v_{2},\ldots v_{n_{v}}\right)}^{T}$represents the state vector for visible layer, where *v*_*i*_ is the state of the *i*th unit in visible layer. $h={\left(h_{1},h_{2},\ldots h_{n_{h}}\right)}^{T}$represents the state vector value for hidden layer, where *h*_*i*_ is the state of the *i*th unit in hidden layer. $a={\left(a_{1},a_{2},\ldots a_{n_{v}}\right)}^{T}$represents the bias vector for visible layer, where *a*_*i*_ is the bias of the *i*th unit in visible layer. $b={\left(b_{1},b_{2},\ldots b_{n_{h}}\right)}^{T}$represents the bias vector for hidden layer, where *b*_*i*_ is the bias of the *i*th unit in hidden layer. $W=\left(w_{i,j}\right)\in {\mathbb{R}}^{n_{h}\times n_{v}}$represents the weight matrix between visible layer and hidden layer, where *w*_*i*,*j*_ is the connecting weight for the *i*th unit in visible layer and the *j*th unit in hidden layer. The units in both hidden and visible layers are binary-valued.

Based on the above notations, the energy function for a given (**v,h**) is defined as:

$$E_{\theta }(v,h)=-a^{T}v-b^{T}v-h^{T}Wv$$ (2)

The joint probability distribution function can be expressed as:

$$P_{\theta }(v,h)=\frac{1}{Z_{\theta }}e^{-E_{\theta }(v,h)}$$ (3)

Where *θ* = (*W*,**a**,**b**), $Z_{\theta }=\sum_{v,h}e^{-E_{\theta }(v,h)}$is defined as the partition function to normalize the probability function.

The conditional probability that hidden unit *h*_*k*_ is activated given hidden vector **v** and the conditional probability that visible unit *v*_*k*_ is activated given visible vector **h** can be respectively calculated as:

$$P\left(h_{k}=1|v\right)=sigmoid\left(b_{k}+\sum_{i=1}^{n_{v}}w_{k,i}v_{i}\right)$$ (4)

$$P\left(v_{k}=1|h\right)=sigmoid\left(a_{k}+\sum_{i=1}^{n_{h}}w_{k,i}h_{i}\right)$$ (5)

*sigmoid* means the sigmoid activation function. Because there is no dependency within visible layer and hidden layer, both conditional probability functions can be written as:

$$P\left(h|v\right)=\prod_{k=1}^{n_{h}}P\left(h_{k}=1|v\right)$$ (6)

$$P\left(v|h\right)=\prod_{k=1}^{n_{v}}P\left(v_{k}=1|h\right)$$ (7)

To find the parameters *θ*, RBM is required to maximize the probability of training set *V*:

$$\arg\underset{\theta }{\max}\prod_{v\in V}P(v)$$ (8)

This is equal to maximize the log likelihood of *P*(v). A common optimization method is gradient descent algorithm. The mathematical form can be expressed in (Equation 9), where the derivative of ln *P*(*v*) should be calculated as (Equation 10) in advance.

$$\theta =\theta +\eta \frac{\partial \ln P(v)}{\partial \theta }$$ (9)

$$\frac{\partial \ln P(v)}{\partial \theta }=-{\langle \frac{\partial E(v,h)}{\partial \theta }\rangle }_{P(h|v)}+{\langle \frac{\partial E(v,h)}{\partial \theta }\rangle }_{P(v,h)}$$ (10)

Where *η* is known as the learning rate, and it is responsible to control the speed of convergence to find the optimal solution. ${\langle \cdot \rangle }_{P}$denotes the expectation value with respect to probability distribution *P*. Solving (equation 8) is computational intensive, Hilton [18] proposed the famous Contrastive Divergence (CD) approach to train RBM in an efficient fashion. The key of this algorithm is to perform *n*-step Gibbs sampling for approximating the expectation value of (Equation 10), the equation can be further simplified into the following three equations:

$$\frac{\partial \ln P(v)}{\partial \theta }=\left\{ \begin{array}{l} \frac{\partial \ln P(v)}{\partial w_{i,j}}\approx P(h_{i}=1|v^{\left(0\right)})v_{j}^{\left(0\right)}-P(h_{i}=1|v^{\left(n\right)})v_{j}^{\left(n\right)}\\ \frac{\partial \ln P(v)}{\partial a_{i}}\approx v_{i}^{\left(0\right)}-v_{i}^{\left(n\right)}\\ \frac{\partial \ln P(v)}{\partial b_{i}}\approx P(h_{i}=1|v^{\left(0\right)})-P(h_{i}=1|v^{\left(n\right)}) \end{array}\right.$$ (11)

The probability distribution function for RBM can be modified as a conditional probability distribution function depending on the previous state of RBM [19], and this leads to a variant of RBM model for efficiently modeling complex time series [20].

### Recurrent Neural Network (RNN)

**Fig 2:**
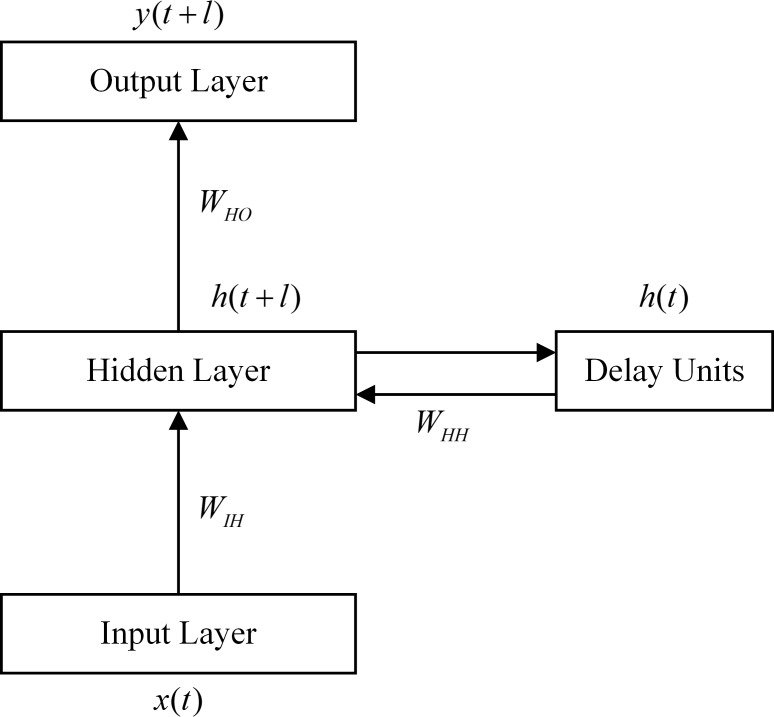
State Space Neural Network Structure.

Recurrent Neural Network (RNN) is a special form of neural network family, and contains at least one feed-back connection as an internal state from neurons’ outputs to their inputs. This loop structure grants the capability of temporal processing and sequence learning for the network. Due to RNN’s short-term memory, it is widely used to model non-linear time series data [21]. Fig. 2 presents a State Space Neural Network (SSNN), which a widely used structure of RNN. Delay units are introduced to feed back the previous hidden unit activations with the inputs into the neural network. *x*(*t*) and *h*(*t*) respectively represent the vectors in input layer as well as delay units at time *t*. *h*(*t*+*l*) and *y*(*t*+*l*) respectively represent the vectors in hidden layer and output layer at time *t*+*l*. *W*_*IH*_ is the weight matrix from input layer to hidden layer. *W*_*HH*_ is the weight matrix from the delay units to hidden layer. *W*_*HO*_ is the weight matrix from hidden layer to output layer. A hidden layer connects both input layer and output layer. The hidden states are delayed by *l* time step, and fed back to the network as additional inputs. This procedure can be described in (Equation 12) and (13).

$$h(t+l)=\sigma (W_{IH}x(t)+W_{HH}h(t))$$ (12)

$$y(t+l)=\sigma (W_{HO}h(t+l))$$ (13)

Where *σ*(•) denote the activation function such as sigmoid function, tangent function and linear function.

**Fig 3:**
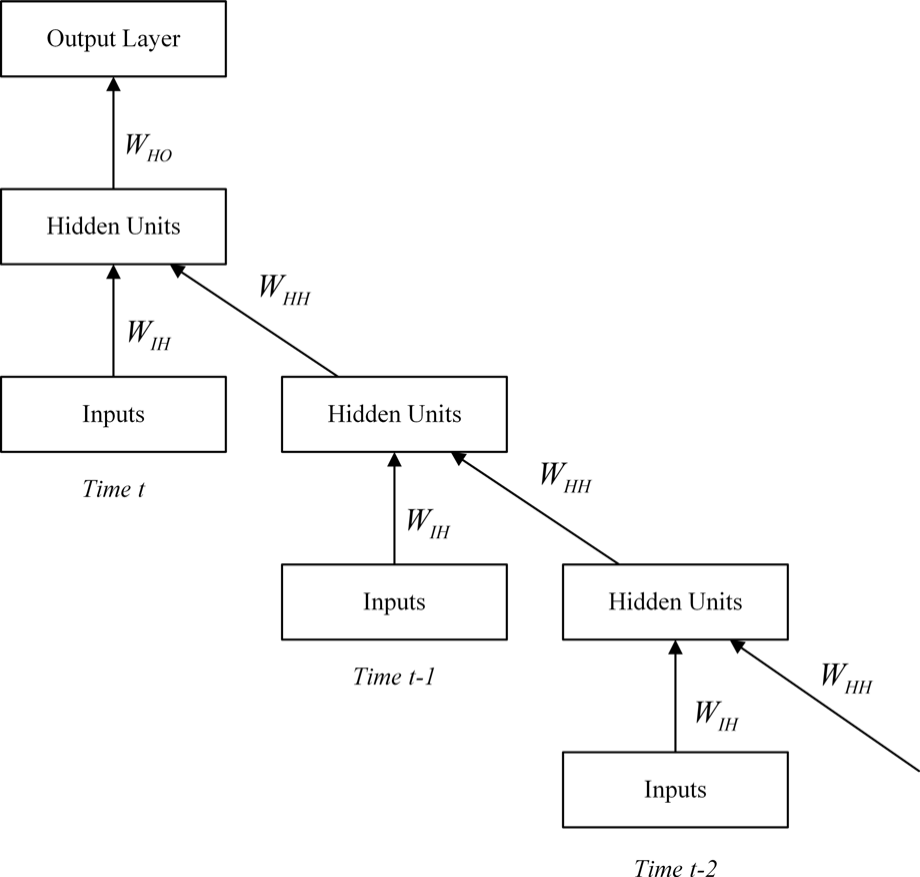
BackPropagation Through Time for RNN.

Training RNN is similar with training traditional multilayer Feed Forward Neural Network (FFNN). By unfolding the RNN structure over time, each feedback loop will be expanded as single layer feed-forward neural network at each time stamp in Fig. 3. In this case, RNN can be efficiently trained using the well-known back prorogation algorithm over time. This is also known as BackPropagation Through Time (BPTT). However, BPTT has difficulty training the sequence with long-term dependency since it can be converted into a deep architecture with multiple layers, and results in vanishing gradient as well as exploding gradient issues [22]. Although there are a certain number of algorithms proposed to solve these issues such as long short term memory network [23], it is still worth investigating in the context of deep learning theory.

### RNN-RBM

**Fig 4:**
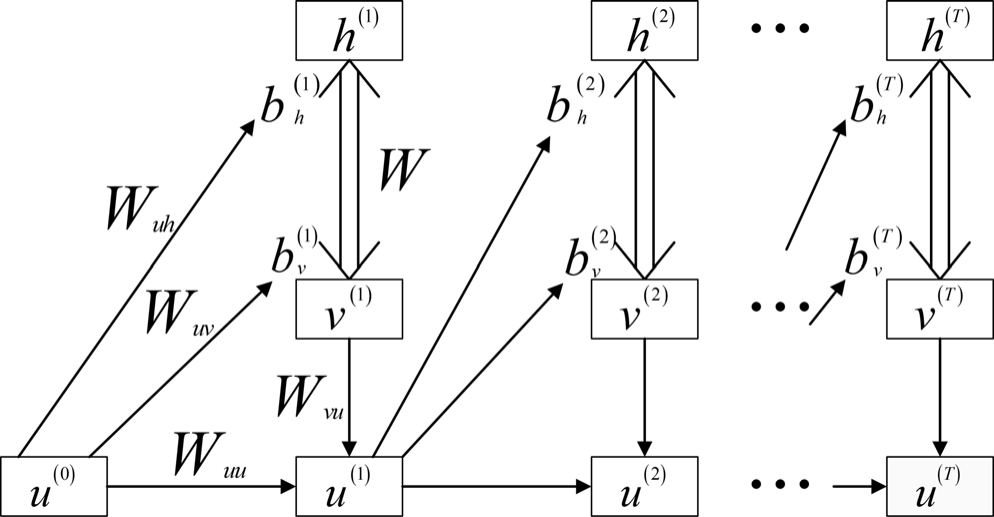
RBM-RNN Architecture.

Both RBM and RNN models have the capability of predicting a temporal sequence. A natural thought is to take advantage of the merits from both models. This triggers a deep architecture called RNN-RBM model to depict the temporal dependencies in high-dimensional sequences [17]. The architecture of RBB-RBM is given in Fig. 4:

The conditional RBM and RNN are stacked to construct a RNN-RBM model. Conditional RBM is an extension of conventional RBM, and it is designed to process temporal sequence by providing a feedback loop between visible layer and hidden layer. The bias values for both visible layer and hidden layer are updated based on the previous visible units [24]. The similar concept can be applied in the RNN-RBM model. $b_{v}^{\left(t\right)}$and$b_{h}^{\left(t\right)}$respectively represent the bias vectors for visible layer and hidden layer in RBM model at time *t*, and are updated through the hidden units *u* ^(*t*-1)^ in RNN model at time *t*-1. Weight matrices *W*_*uv*_ and *W*_*uh*_ are provided to connect the RNN model and RBM model. The above procedure can be described in (Equation 14) and (Equation 15).

$$b_{v}^{\left(t\right)}=b_{v}+W_{uu}u^{\left(t-1\right)}$$ (14)

$$b_{h}^{\left(t\right)}=b_{h}+W_{uh}u^{\left(t-1\right)}$$ (15)

Where *b*_*v*_ and *b*_*h*_ are the initial biases in visible layer and hidden layer for RBM model. The RNN model is unfolded over time, and is utilized to generate the previous hidden states in RBM model based on the input layer *v* ^(*t*)^ and hidden layer *u* ^(*t*)^ in RNN model. The activation of hidden units in the hidden layer can be then calculated as:

$$u^{\left(t\right)}=sigmoid(b_{u}+W_{uu}u^{\left(t-1\right)}+W_{vu}v^{\left(t\right)})$$ (16)

The algorithm execution is summarized below:

*Step 1*: Generate the value of hidden units in RNN model using (Equation 16).

*Step 2*: Update the biases in the RBM model using (Equation 14) and (15) based on the estimated *u* ^(*t*-1)^ in *Step 1*, and calculated the RBM parameters.

*Step 3*: Calculate the log-likelihood gradient $\frac{\partial \ln P(v)}{\partial \theta }$ in RBM model using (Equation 11)

*Step 4*: The estimated gradient is propagated into RNN model, and the weight *W*_*uv*_ and *W*_*vu*_ are updated over time to train RNN model for prediction.

### Temporal-Spatial Congestion Evolution Prediction

As previously mentioned, traffic condition on each roadway segment can be converted into a binary value denoting congestion status. Considering historical traffic patterns and number of links in a large-scale traffic network, the congestion evolution prediction problem becomes a high-dimensional temporal sequence learning problem. The RNN-RBM model can be applied to predict the temporal-spatial network congestion evolution pattern. To implement the RNN-RBM model in an efficient manner, the mini-batches gradient descent optimization method is applied: the training set is divided into a number of small samples, and each sample set can be parallelly processed using standard gradient descent optimization method. GPU is designed on a parallel throughput architecture that can execute multiple tasks concurrently [25]. This feature is especially suitable to accelerate the computation of RNN-RBM model. CUDA is a parallel computing platform developed by NVIDIA [26], and it offers a flexible programming library for users to manipulate the computational elements in GPUs.

## Numerical Study

### Model Development

To validate the effectiveness and efficiency of the proposed network-wide congestion prediction approach, a transportation network in Ningbo City, China is used for the numerical experiment. The network consists of 515 road links where approximately 4000 GPS-equipped taxis traveled from April 13, 2014 to May 9, 2014. The GPS data are updated every 2 minutes. A series of data quality control methods are applied to remedy the missing or erroneous data, and this generates 5,521,294 GPS records with speed information. Each GPS record is associated with a unique link ID, and this link ID corresponds to a specific geospatial polyline in a GIS platform. These GPS data are further aggregated into 5 minutes, 10 minutes, 30 minutes and 60 minutes time intervals to evaluate the network traffic congestion prediction performance. The data from the first 22 days (i.e. from April 13, 2014 to May 4, 2014) are utilized for training the RNN-RBM model, and the remaining data are used for testing.

Minimizing cross-entropy error is set as the optimization objective during the RNN-RBM model training procedure. This is because that the cross-entropy indicates the distance between the probability distributions of computed outputs and target outputs, and thus it is preferable to using mean squared error for a neural network classifier with binary values [27]. The cross-entropy error is defined in (Equation 17).

$$\text{Cross-Entropy Error (CEE)=-}\frac{1}{T}\sum_{n=1}^{N}\sum_{t=1}^{T}c_{n}^{t}\ln({\hat{c}}_{n}^{t})+(1-c_{n}^{t})ln(1-{\hat{c}}_{n}^{t})$$ (17)

In addition, both training accuracy and testing accuracy are presented to measure the algorithm effectiveness. The estimated congestion status for link *n* at time *t* is denoted as${\hat{c}}_{n}^{t}$. According to (Equation 1), the ground-true congestion status for link *n* at time *t* is denoted as$c_{n}^{t}$. Because both ${\hat{c}}_{n}^{t}$ and $c_{n}^{t}$ are binary values, the prediction accuracy can be defined in (Equation 18) given a transportation network with *N* links within *T* time intervals:

$$\text{Accuacy (Acc)=(1-}\frac{\sum_{n=1}^{N}\sum_{t=1}^{T}\left|c_{n}^{t}-{\hat{c}}_{n}^{t}\right|}{NT})\times 100\%$$ (18)

If a positive result refers to each link that is correctly identified as either congested or uncongested, then the sensitivity and specificity can be respectively defined as:

$$\text{Sensitivity=}\frac{\text{number of links correctly identifed as congested}}{\text{total number of congested links}}$$ (19)

$$\text{Specificity=}\frac{\text{number of links correctly identifed as uncongested}}{\text{total number of uncongested links}}$$ (20)

Algorithm runtime is used to evaluate the efficiency of the proposed approach.

Parameters in the RNN-RBM model are set optimally as follows:

Number of hidden units in the RNN model = 100

Number of hidden units in the RBM model = 150

Learning rate for gradient descent optimization method = 0.05

The weight matrix *W* for RNN-RBM model is initialized by following a Normal distribution with mean = 0 and variance = 0.01, and other matrices *W*_*uu*_,*W*_*uv*_,*W*_*vu*_ and *W*_*uh*_ are initialized by following a Normal distribution with mean = 0 and variance = 0.0001. All the bias vectors in the model are set as zero initially.

### Result Analysis

GPS speed data with four different aggregation levels were tested using the RNN-RBM model, and the trained model was then utilized to generate the future network-wide congestion evolution patterns from May 5, 2014 to May 9, 2014 for prediction. Each model was run for 200 iterations to ensure the algorithm’s successful convergence. The algorithm was implemented using Python Theano [28] and was executed on a desktop computer with Intel i7 3.4GHz CPU, 8GB memory and NVIDIA GeForce GTX650 GPU (2GB RAM). The comparison results are demonstrated in Table 1:

**Table 1:** Comparison of traffic congestion prediction performance with different data aggregation levels.

Aggregation Level	Training Accuracy (%)	Cross-Entropy Error	Runtime (seconds)	Testing Accuracy (%)
60 minutes	95.1	-38.7	354	88.2%
30 minutes	88.4	-102.52	643	80.8%
10 minutes	80.2	-172.3	1729	73.4%
5 minutes	72.2	-239.8	4642	68.9%

Results in Table 1 demonstrate that the overall algorithm prediction performance improves as the data aggregation level increases. This is probably due to the less data fluctuation (i.e. traffic condition does not change dramatically) as the time interval becomes longer. When the aggregation level achieves to one hour, the training accuracy is 95%, and more than 88% of congestion conditions can be correctly identified, while the algorithm can be successfully executed within 6 minutes. Both testing accuracy and training accuracy degrade when the data resolution becomes higher. However, the sensitivity and specificity do not straightly increase as the data aggregation level becomes larger. For instance, only 43% of congested linked can be correctly predicted when the aggregation level is set as 10 minutes. This implies that the data aggregation level influences the prediction outcomes, and should be carefully selected.

In addition, CUDA-based GPU parallel computing engine presents its superior capability of concurrently accelerating the computational intensive tasks. Compared with the CPU-based algorithm implementation, the proposed GPU-based framework can approximately improve the algorithm efficiency by 5 times.

**Fig 5:**
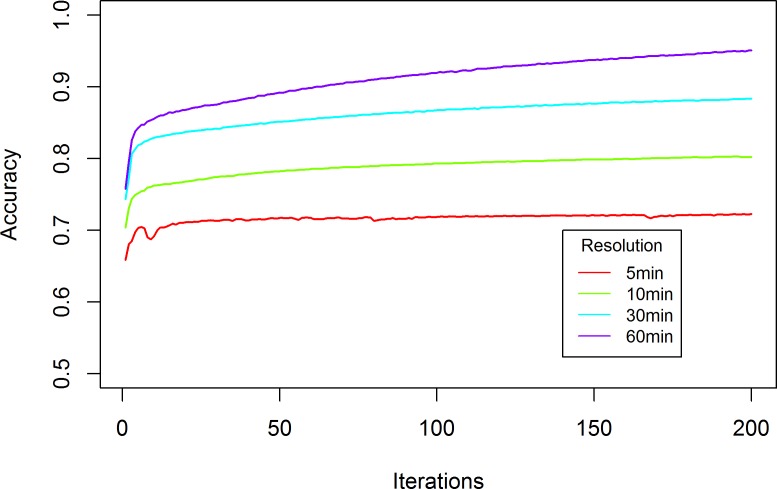
Training Accuracy Changing Curves with Different Data Aggregation Levels.

The training accuracy changing curves for the above four scenarios are plotted in Fig. 5:

RNN-RBM models with varying data aggregation levels can converge after 150 iterations, and the training accuracy remains almost constant. This indicates that the RNN-RBM model is able to learn the network-wide traffic congestion patterns in a fast and reliable fashion.

### Visualization

**Fig 6:**
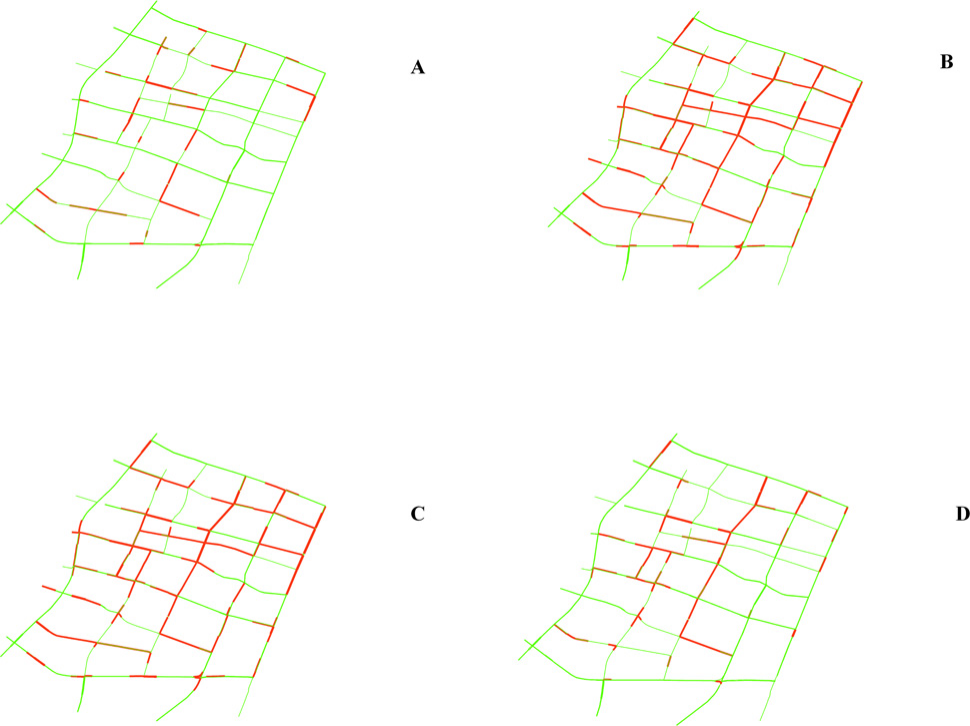
Predicted Network Congestion Evolution Patterns on May 09, 2014 with Varying Times of Day. (a) Spatial Distribution of Congestion from 5AM to 6AM; (b) Spatial Distribution of Congestion from 9AM to 10AM; (c) Spatial Distribution of Congestion from 5PM to 6PM; (d) Spatial Distribution of Congestion from 11PM to 12PM (Red line indicates congested traffic condition; green line indicated uncongested traffic condition).

Based on the trained RNN-RBM models, the network congestion evolution patterns from May 5, 2014 to May 9, 2014 can be thereby predicted. As previously mentioned, each binary congestion status can be mapped on a GIS platform using the unique link ID. To visualize the spatial and temporal network congestion onset and growth, a series of dynamic GIS networks with colored links were created in Fig. 6A-Fig. 6D based on the predicted traffic congestion patterns on May 9, 2014.

The congested link (less than 20 km/hour) is marked in red, and the uncongested link (equal to or greater than 20 km/hour) is marked in green. The traffic condition in the early morning was generally good with only a small number of congested links, and the network became more and more congested during morning peak hours. Similarly, when most people commuted during evening peak hours, network-wide traffic congestion reached highest. Finally, traffic jam dissipated at midnight since the majority of passengers engaged in less or no activities during that time. As presented in Fig. 6A-Fig. 6D, traffic congestion initially concentrated in the central of network, and gradually spread to the network boundary. There are several links that consistently experience traffic congestion, and should be taken special attention for congestion mitigation in advance.

**Fig 7:**
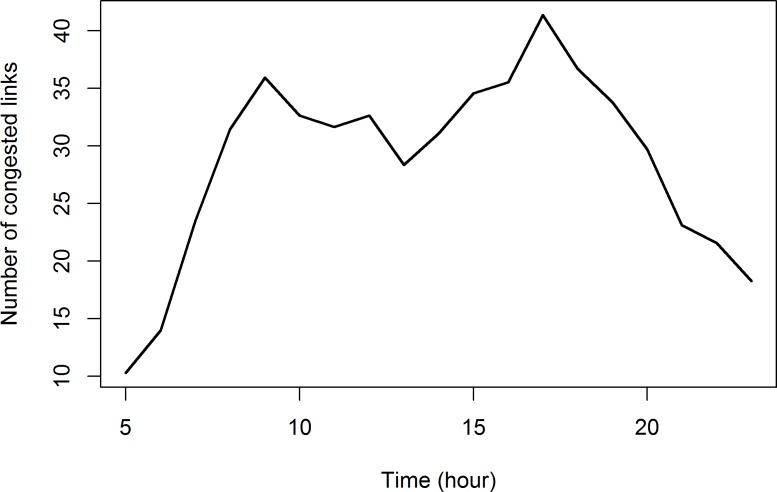
Temporal distribution for number of congested links on May 9, 2014.

The temporal distribution for number of congested links is summarized in Table 2 and visualized in Fig. 7. There are 35.9% of road links experiencing traffic congestion during morning peak hour (9:00AM-10:00AM), while 41.1% of network was congested during evening peak hour (5:00PM-6:00PM). This indicates that the evening peak traffic is worse than morning peak traffic, and causes more delay. People may tend to return home earlier than usual week days for weekend activities, and thus higher traffic volumes on Friday’s evening peak hours can be observed.

**Table 2:** Statistics for number of congested links on May 9, 2014.

Time	Number of congested links	Percentage (%)
5:00–6:00	53	10.3
6:00–7:00	72	14.0
7:00–8:00	121	23.5
8:00–10:00	162	31.5
9:00–10:00	185	35.9
10:00–11:00	168	32.6
11:00–12:00	163	31.7
12:00–13:00	168	32.6
13:00–14:00	146	28.3
14:00–15:00	160	31.1
15:00–16:00	178	34.6
16:00–17:00	183	35.5
17:00–18:00	213	41.4
18:00–19:00	189	36.7
19:00–20:00	174	33.8
20:00–21:00	153	29.7
21:00–22:00	119	23.1
22:00–23:00	111	21.6
23:00–00:00	94	18.3

### Comparison

To further evaluate the advantages of RNN-RBM algorithm for large-scale transportation network congestion prediction, a study was conducted by comparing RNN-RBM, Back Propagation Neural Network (BPNN) and Support Vector Machine (SVM) methods. To remain a fair comparison environment, the same dataset and computing platform was adopted. The topology for BPNN was set as one input layer, one hidden layers with 10 processing units and one output layers\. For the SVM method, Radial Basis Function (RBF) was utilized with three adjustable parameters: cost *C*, width parameter *g*, and epsilon *ε*. These parameters were calibrated using 5-fold cross validation. The rolling horizon (i.e. the size of moving window defining previous network congestion statuses) was set as 2 time steps. To eliminate the randomness, both BPNN and SVM were executed for 10 times. Table 3 presents the performances for three algorithms with 60-minute data aggregation level.

**Table 3:** Comparison of traffic congestion prediction performance for different algorithms with 60-minute data aggregation level.

Algorithms	Runtime (seconds)	Prediction Accuracy (%)	Sensitivity (%)	Specificity (%)
RNN-RBM	354	88.2%	64.1%	91.1%
BPNN	13498	69.7%	38.2%	77.5%
SVM	14979	71.0%	36.6%	80.3%

Due to the limit of computational resources, only 60-minute data aggregation level is utilized for testing. As demonstrated in table 3, the RNN-RBM outperforms the other two algorithm in terms of efficiency and effectiveness: The algorithm execution time for RNN-RBM is only 3% of that for BPNN, and 2.3% of that for SVM. The gain in computational efficiency for RNN-RBM does not sacrifice the algorithm accuracy. Compared with BPNN and SVM, the prediction accuracy for RNN-RBM increases by at least 17%. Moreover, both sensitivity and specificity for RNN-RBM are significantly higher than those for BPNN and SVM. This is owing to the deep and parallel architecture of RNN-RBM for learning multidimensional features in an efficient and effective manner.

### Sensitivity Analysis

Congestion identification relies on the setting of speed threshold when traffic congestion occurs. To understand how various speed thresholds influence the congestion pattern prediction performance, a sensitivity analysis was conducted to examine the prediction capability of RNN-RBM model. Three different speed settings were adopted: 10 minutes, 20 minutes and 30 minutes. When the calculated average GPS link speed is lower than the specified speed threshold, the link is considered congested. The GPS speed data were aggregated into 60-minute interval, and followed the similar model training and testing procedure in the model development. The results are presented in table 4:

**Table 4:** Sensitivity analysis of congestion evolution prediction performance with various speed thresholds.

Speed Threshold	Number of Congested Links	Training Accuracy (%)	Runtime (seconds)	Testing Accuracy (%)
10 km/hour	1895	98.6%	290	93.8%
20 km/hour	13611	95.1%	354	88.2%
30 km/hour	20238	84.0%	419	79.9%

There are a total of 59,225 traffic conditions from May 5, 2014 to May 9, 2014 for GPS data with 60-minute aggregation level. When the speed threshold is set as 10 km/hour, only 1895 congested links can be detected. Surprisingly, the model training and testing accuracies are not significantly higher than those with 20km/hour speed threshold, even though the number of congested links with 20km/hour speed threshold is approximately 7 times more than that with 10km/hour speed threshold. However, when the speed threshold increases to 30 km/hour, more than 30% of network links are congested. The training accuracy decreases to 84%, which is much lower than the scenario with 20 km/hour speed threshold. This is probably because that traffic congestion temporal patterns fluctuate more significantly as the speed threshold is higher, and thus incurs difficulties in predicting congestion evolution. In practice, the setting of congestion speed threshold should be carefully determined.

## Conclusion

Properly understanding the temporal and spatial patterns of congestion evolution is crucial to effectively mitigate congestion. This study proposed a data-driven method to predict the network traffic congestion onset and evolution patterns. Based on the tremendous GPS taxi speed data, the network congestion onset, propagation and dissipation can be modeled using a deep RNN-RBM architecture. To the best of our knowledge, this is the first study to utilize the deep learning theory into large-scale transportation network analysis. Compared with the traditional equation-driven or simulation-driven network studies, the proposed method relies on less assumption to model traffic congestion dynamics. A numerical experiment in Ningbo, China is conducted to validate the effectiveness and efficiency of the proposed method. A transportation network with 515 links is constructed, where the speed information generated from 4000 taxis are mapped and converted into a binary value to represent traffic congestion for each link. The RNN-RBM model has the capability of learning high-dimensional temporal sequences in an accurate and efficient manner, and is implemented in a CUDA-based parallel environment to accelerate the computational procedure. The training accuracy can achieve as high as 95.1% using the GPS data with one hour aggregation level while the algorithm execution time is only less than 6 minutes, and the trained model can be utilized to correctly predict network-wide traffic congestion on more than 88% links. The inferred network congestion patterns are further visualized on a GIS-based map platform to investigate the temporal and spatial evolution of traffic congestion. By comparing with conventional data mining approaches (i.e. BPNN and SVM), the RNN-RBM algorithm demonstrates its superiority in terms of effectiveness and efficiency. In addition, a sensitivity analysis is conducted to understand how different speed thresholds impact the congestion prediction accuracy. The finding of this study is important for large-scale roadway network planning, operations, and investment decisions. Understanding which congested locations are autonomously generated and how congestion propagates over transportation network will allow researchers and practitioners to focus the limited resources on the primary congestion locations, and adopt the proactive countermeasures to mitigate congestion.

Although the proposed method is promising to model and predict large-scale transportation network congestion, there is still plenty of room to be improved in the future research. For instances, the pretraining techniques such as Hessian-free optimization method should be considered to initialize the parameters more rationally. In addition, the spatial interaction among the adjacent roadway segments should be taken into account to increase training and prediction accuracy.

## Supporting Information

S1 Dataset The dataset includes the raw data used for training and testing with data aggregation levels of 30 minutes and 60 minutes. The data collection period is from April 13, 2014 to May 9, 2014. Data from April 13 to May 4 are utilized for training models, and the remaining data are utilized for testing and prediction. For each CSV (Comma-Separated Values) file, the first row indicates the road link IDs in the entire network, and the other rows indicates the traffic conditions for each time interval, where 0 represents uncongested and 1 represents congested. The congestion threshold is set as 20 kilometers per hour. The algorithm execution results are saved in two CSV files. The file named as “result.csv” records both accuracy and cross-entropy value for each iteration, and the other file named as “sequence.csv” records the predicted traffic congestion patterns using the RBM-RNN model.
(ZIP)
